# COVID-19 contact tracing apps: UK public perceptions

**DOI:** 10.1080/09581596.2021.1909707

**Published:** 2021-04-23

**Authors:** G. Samuel, S. L. Roberts, A. Fiske, F. Lucivero, S. McLennan, A. Phillips, S. Hayes, S. B. Johnson

**Affiliations:** a Department of Global Health and Social Medicine, King’s College, London, UK; b Institute for Global Health, University College London (UCL), London, UK; c Institute of History and Ethics in Medicine, Technical University Munich, Munich, Germany; d Ethox Centre, University of Oxford, Oxford, UK; e Institute for Biomedical Ethics, University of Basel, Basel, Switzerland; f Centre for Biomedical Ethics and Law, Department of Public Health and Primary Care, KU Leuven, Leuven, Belgium; g Vienna School of International Studies, Diplomatische Akademie Wien, Vienna, Austria

**Keywords:** Contact-tracing apps, COVID-19, digital surveillance, pandemic, qualitative research, responsible innovation, anticipatory governance

## Abstract

In order to combat the COVID-19 pandemic, policymakers around the globe have increasingly invested in digital health technologies to support the ‘test, track and trace’ approach of containing the spread of the novel coronavirus. These technologies include mobile ‘contact tracing’ applications (apps), which can trace individuals likely to have come into contact with those who have reported symptoms or tested positive for the virus and request that they self-isolate. This paper takes a critical public health perspective that advocates for ‘genuine participation’ in public health interventions and emphasises the need to take citizen’s knowledge into account during public health decision-making. In doing so, it presents and discusses the findings of a UK interview study that explored public views on the possibility of using a COVID-19 contact-tracing app public health intervention at the time the United Kingdom (UK) Government announced their decision to develop such a technology. Findings illustrated interviewees’ range and degree of understandings, misconceptions, and concerns about the possibility of using an app. In particular, concerns about privacy and surveillance predominated. Interviewees associated these concerns much more broadly than health by identifying with pre-existent British national narratives associated with individual liberty and autonomy. In extending and contributing to ongoing sociological research with public health, we argue that understanding and responding to these matters is vital, and that our findings demonstrate the need for a forward-looking, anticipatory strategy for public engagement as part of the responsible innovation of the COVID-19 contact-tracing app in the UK.

## Introduction

This paper is concerned with taking a critical public health perspective that advocates for ‘genuine participation’ in public health interventions and emphasises the need to take citizen’s knowledge seriously into account during public health decision-making (Green, 2006). To do this, the paper reports the findings of an in-depth UK interview study which explored public views on the possibility of using the public health intervention, a COVID-19 contact tracing application (app), at the time the UK government announced their decision to develop such a technology to help mitigate the spread of the coronavirus.

This paper also contributes to sociological research that engages with public health (Mykhalovskiy et al., 2019), as it is concerned with the need for a forward looking, anticipatory (Guston, 2013) public engagement as part of the responsible innovation of the COVID-19 app. Responsible innovation, which is a vital aspect of contemporary sociological and science and technology studies (STS) literature and policy, recognises the importance of embedding responsibility and integrity into the governance of emerging technologies such as contact tracing apps, through the anticipation and response to ethical, social and political dimensions associated with their societal impact (Stilgoe et al., 2013). It views research as a collective endeavour, such that engagement across all stakeholders is an integral aspect of the innovation and governance process. In particular, and of relevance for this paper, an important constituency in responsible innovation, and representing one of the stakeholder groups whose participation should be sought and facilitated, are members of the public (Lehoux et al., 2020). Understanding the views of members of the public, and responding to them is seen as vital, since it is the public who *‘give warrant to the development of innovations as citizens and taxpayers and who may be exposed to their benefits and risks as users’* (Lehoux et al., 2020). As part of responsible innovation, such views can contribute to an anticipatory governance by allowing previously unknown challenges that have been exposed through public engagement to be confronted and integrated into innovation governance (Lehoux et al., 2020).

COVID-19 contact tracing technologies can identify individuals likely to have come into contact with those who have reported symptoms or tested positive for the virus and request that they self-isolate (WHO, 2020). In April 2020, the UK government announced plans to develop a contact tracing app that would be key to exiting the March 2020 lockdown during the first wave of the pandemic. The aim was to deploy the app within weeks of its announcement, with the expectation that the technology would rapidly render patterns of infection visible to public health authorities and would lead to targeted public health interventions in reducing the spread of new cases of COVID-19. An April 2020 UK survey found that while three quarters of those surveyed were confident that they could download and use a contact tracing app, only around 40% indicated that they were confident in the ability of the government to protect personal data (Duffy, 2020). Other surveys conducted at the same time found similar support for contact tracing apps, but with a quarter of participants feeling anxious about government surveillance after the pandemic (Abeler et al., 2020). These figures were important: contact tracing apps require broad public support in order to work as intended, with an estimated necessary adoption threshold of approximately 60%.^1^ Addressing public concerns related to contact tracing apps is therefore essential for apps to be useful public health tools for combating the spread of COVID-19 and future outbreaks.

This paper approaches these survey findings from a critical perspective that understands the limitations of mechanistic surveys to be able to provide insight into why people hold particular views, something that is vital when implementing any public health intervention (Green, 2006). Using qualitative methods, which are designed to understand the reasons why people hold certain views, the aim of this paper is to contribute to a responsible innovation of the UK contact tracing app by exploring UK public understanding and perceptions of digital contact tracing apps early in the technology’s innovation process, during the period that the UK first announced its plan to develop and implement such a technology. By doing so, and by better understanding the reasons and justifications behind public perceptions about the app at an early stage, the aim of the paper is to contribute to anticipatory governance of the technology. More broadly, this paper works to bring the concept of responsible innovation to the public health arena, as a useful governance mechanism for public health interventions that are synonymously emerging technologies, such as contact tracing apps.

Many concerns were raised about the possible use of such an app, including issues related to privacy, surveillance, technical capacity and equity (see below). Scholars and commentators alike called for appropriate oversight, institutional responsibility and transparency as part of the UK contact tracing app innovation process (Lucivero et al., 2020; NHSX App Ethics Advisory Board, 2020; Parker et al., 2020), especially given that much of the support for digital contact tracing apps, or lack thereof, seemed to be strongly influenced by public trust in the UK government and other institutions (AdaLovelace Institute, 2020a).

## Background

In April 2020, the British public was informed that NHSX, the unit of the National Health Service (NHS) responsible for digital innovation, and the unit responsible for the delivery of the app, had been working with private companies and researchers from Oxford University to develop a digital contact tracing tool. The UK app, similar to other European countries, would rely on Bluetooth-based technology, which is deemed more privacy-preserving compared to the use of GPS-based app technology that has been used in other countries, such as Taiwan and South Korea (Huang et al., 2020; Steinbrook, 2020). This technology allows the collection of random codes that are shared by devices in close proximity for a specific length of time. When a positive case is registered on a device’s app, all devices which have recently shared a code via Bluetooth will receive an alert to self-isolate. In this model, when app users alert the app that they may have COVID-19 symptoms, data about other devices a user has been in contact with are transmitted back to a central anonymised database. The centralised model of data collection had the benefit of being able to follow the spread of reported symptoms between contacts, allowing public health authorities to: identify high risks individuals or groups, to predict the cases of reported symptoms that will result in a positive test, and to follow up with people who are self-isolating to let them know if they need to continue for the full 14 days (Paul & Irvine, 2020). This model had been prominently criticised by many who see it as ‘privacy diminishing’. Concerns include risks posed by the collection, use, and storage of personal data by a digital tool (DP-3T project, 2020; Lucivero et al., 2020), as well as concerns about the contact tracing apps being re-appropriated to introduce and normalise the increased, automated and routinised population surveillance for purposes beyond containing the spread of infectious diseases (AdaLovelace Institute, 2020b; Amnesty International, 2020; Gasser et al., 2020; Kitchin, 2020; Nikel, 2020; Wienroth et al., 2020). Concerns had also been raised about the nature of the NHSX-Tech company collaborations in terms of who would have access to data collected by the app, and under what conditions, including concerns that data gathered could be fed into the NHS COVID-19 datastore, which involves large technology (‘Big Tech’) partners such as Google, Amazon and Palantir (Gould et al., 2020).

As such, some scholars advocated for a ‘decentralised’ approach to digital contact tracing based on an API^2^ provided by Google and Apple, in which random codes shared by devices typically remain on people’s phones. Technology companies argued de-centralised data collection and storage makes it harder for hackers or the authorities to trace and identify specific individuals and is therefore privacy enhancing. But this raised further issues regarding the increased involvement of Big Tech corporations in public health solutions (for example, see Johnson et al., 2020; Lucivero et al., 2020; Sharon, 2020; Roberts, 2020a).

Concerns more generally about the use of a contact tracing apps also included various feasibility and technical issues, including those of interoperability with apps developed in other countries, and the fact that the app would not work on a variety of older smartphones (The Health Foundation, 2020; Wright, 2020); concerns that the process of consent to use the app may not ensure understanding (Bengio et al., 2020; Raskar et al., 2020); and that app usage could reinforce digital divides, exacerbate health inequalities, or unfairly discriminate against particular groups (AdaLovelace Institute, 2020a; French et al., 2020; Gasser et al., 2020; Morley et al., 2020; NHSX App Ethics Advisory Board, 2020).

## Methods

### Data collection

This research is part of a multinational (based on nine European countries) qualitative longitudinal interview study on ‘Solidarity in times of pandemics’ at the University of Vienna (SolPan Consortium^3^). In the UK, 35 semi-structured qualitative interviews were conducted between the 6th-30th April 2020 (early during lockdown). Interviews took place online or via telephone, and were audio recorded and transcribed. A researcher–developed interview guide was used to guide discussion, including asking participants about their knowledge and views of any new technologies that could help contain the spread of the virus. During the interviews, interviewers encouraged reflection about interviewees’ knowledge and views on new technologies and contact tracing apps. If interviewees had not heard of contact tracing apps, they were provided with a brief explanation of their purpose and function as a response tool during the first wave of the pandemic in the UK. Discussions with interviewees regarding contact tracing apps remained topical and descriptive and did not focus on overly technical explanations of the app or underlying technologies (such as GPS or Bluetooth). Interviewees were then asked their opinions, with interviewers probing any ambivalence or contradictions. Interviews were between 30–65 minutes. Demographics of interview participants are reported in Table 1.

### Data analysis

At the SolPan Consortium level, each member of the SolPan ‘data analysis team’, which included at least one member from each contributing country, independently coded the same interview transcript, the insights from which were formulated into a draft coding scheme. This draft scheme was independently applied to a country-specific sample transcript by each member of the analysis team. Findings were compared, and a project-wide inductively generated coding scheme that could be applied to all interviews was produced. At the UK level, interview transcripts were coded by all authors of the paper using the standardised coding scheme, with the assistance of the atlas.ti software. Following this, lead authors [GS/SR] carefully read and re-read the relevant codes of all interview transcripts, discussed these codes, and generated preliminary themes emerging from the data drawing on their knowledge of public health ethics. Preliminary themes were then presented and discussed with all authors. An iterative process of development produced final higher order themes, presented below.

### Ethics

Ethical approval for the study was obtained from the University of Vienna Ethics committee Reference Number 00544.

## Findings

### Awareness of digital contact tracing apps and perceived benefits and risks

Many interviewees reported that they had not heard of contact tracing apps at the time of interview in April 2020, or that they were minimally aware of such apps, but had very little understanding of them. For those interviewees who had heard of apps (and for those who had not, but for whom interviewers described their underlying rationale), many were supportive of the idea of an app to help control the spread of the virus *(‘that sounds amazing’* (interviewee 26)). For those who supported the use of the app, participants provided a range of different rationales for their support. Interviewee 27 drew on well-versed arguments about the intrinsic value of big data research to enthusiastically support the use of an app, explaining how all health data should be placed in an NHS database for research purposes; *‘everybody’s information should be on the NHS system … to help research … to help humankind go forward’.* More prominently, interviewees felt the use of an app could help contain the virus, and act as a key necessity for easing lockdown by helping *‘people [to be] allowed to go back to work’* (interview 5), or, for others, assist them with going *‘out and [to] have a slightly more normal life’* (interview 9). For a few interviewees who were particularly concerned about the virus and the risk it posed to society, the potential benefits brought by any app in helping to control the spread of the virus was so great that it was proportional for individuals to give up key liberties relating to being able to choose whether or not to use such an app; *‘I’m hoping they’re sensible enough to say, “look you know everybody who’s got a smartphone must use it”. It would really be the only way it would work’* (interviewee 28; emphasis added).

At the same time, interviewees had varying beliefs as to the potential for these benefits to be realised, and the power of an app to take a central role in controlling the pandemic. Interviewees raised concerns about the feasibility and perceived social or practical limitations of contact tracing apps associated with adherence, misuse, and behaviour (e.g. individuals forgetting to take phones with them), and some indicated a lack of understanding of how to download any potential app or use it effectively. Interviewees also had concerns about the technicalities and ‘functionality’ of apps. Going beyond what would normally be identified in survey data, interviewees spoke about the difficulties with weighing up different values. For example, the permissibility of a potential UK app became difficult to balance when interviewees considered the range of associated potential harms that came with using apps. Most prominently, this related to worries associated with infringements of privacy and surveillance (‘I’m *a bit reluctant to take it up … it’s hard to know. It’s one kind of freedom for another kind of freedom’* (interviewee 1)). For many of our interviewees, their support for digital contact tracing apps then became a balancing act – weighing up, on the one side, infringements of privacy, with, on the other side, the potential benefits of using an app. The positions interviewees took in these deliberations were particularly influenced by how much they felt digital contact tracing apps would infringe on privacy and surveillance. It was these issues that predominated much of interviewees’ discussions about apps. Below, we explore views on privacy and surveillance in more detail, paying particular attention to how their views related to their understanding of digital contact tracing apps, and to their broader views and beliefs about the UK political context more generally.

### Privacy, liberty and surveillance

Many interviewees’ concerns about using contact tracing apps were framed around the belief that the technology could ‘track’ individuals, which for many, was an important and sometimes deciding factor in whether or not they supported the use of apps as a containment tool during the pandemic.

When interviewees explained their perceptions of what ‘tracking’ meant to them, different understandings of the term emerged. Most strikingly, and highlighting misperceptions of digital contact tracing apps, the element of personal reflection was particularly interesting: interviewees constructed a picture of tracking, quite literally, as functioning at the individual level, in which they imagined an individual being able to ‘see’ or ‘visualise’ their every move. They spoke about being identified when they were ‘in the middle of a field’ or joked about being tracked in their houses, and during their shifts at work:

If they see a mobile phone in the middle of a field, how do they know that person is not a farmer? (interviewee 3);

I mean, if they track me at night-time when I’m working, they’ll find I’m here, there, everywhere. So, they’d be like, where on Earth is she going? (interviewee 2; our underline);

I think they’d be bored trying to check me around the house (interviewee 8).

Several interviewees also spoke about any potential app being employed as a way to alert the authorities if an individual broke lockdown rules. Interviewee 30, for example, compared their conceptualisations of the role of any contact tracing app with that of law enforcement apprehending those individuals who need to be ‘caught’:

I just think that’s government going into our privacy now if they use that [an app] … they’ve got the police out there anyway, so if they catch you, they catch you. But I don’t think they should be going to check people through the phones …

Interviewee 24 also reflected on the fact that the data collected through some apps could be analysed in such a way as to determine whether or not they had left the house when they were supposed to:

You don’t need to know where people have been … and why they’ve been there … Obviously you’re not meant to go out and do anything, but I’ve had to take a few things to my nan’s house and stuff.

It was perhaps because of interviewees’ misunderstanding of digital tracing apps as geo-location trackers, or maybe in spite of it, that some interviewees’ raised concerns about the potential for data collection through the apps, and subsequent data analysis, to be used in such a way that would lead to the increased surveillance of society. Though views were mixed: while some interviewees were concerned about the prospect of surveillance, others were less concerned. Nonetheless, all interviewees were well versed in the general concept of surveillance and could narrate it well irrespective of their opinion on contact tracing apps, and nearly all articulated the issues at stake for contact tracing apps in terms of the wider Big Brother narrative, in which interviewees pointed towards an overly-controlling authority that could invade the privacy of citizens’ lives.

For those uncomfortable with the idea of what they perceived as being tracked by the private sector or the UK government, they were less supportive of using apps and more concerned about the prospect of surveillance through the use of apps. Common metaphors were used to describe their views. Some interviewees used the term ‘Orwellian’ – a reflection of how the term Big Brother originally emerged from George Orwell’s *1984* book, others analogised their use to the science fiction movie *Blade Runner,* referencing a dystopian world mediated by androids, robotics and cybernetics:

it’s a whole double-edged sword, isn’t it? On the one hand, yes, technology to try and contain something horrendous. Brilliant. On the other hand, how flipping Orwellian is that? (interviewee 16);

it’s quite scary. I’m thinking [of] Blade Runner. It’s a difficult one, isn’t it? It’s a real moral dilemma (interviewee 15).

For these interviewees, the use of an app was not considered as a discrete or independent endeavour, but for some, sat within their broader concerns about the prospect of surveillance in society more generally – which some interviewees had expressed that they have been fighting against for many years – while others associated contact tracing apps as a potential encroachment on liberties and freedoms. The app, in this sense, was used as a hook for their broader concerns about infringements of privacy. For example, some interviewees contextualised the app within discourses of *‘individual liberties and responsibilities’* they had *‘worked so hard for’* in the past:

these are the extraordinary questions, aren’t they? That we are now having to face where we’ve always been so used to, we worked so hard for liberty, individual liberties and responsibilities and so on (interviewee 17).

Interviewee 8 provided the example of identity cards to highlight their concerns about surveillance:

I don’t trust that surveillance. We don’t have identity cards, but everybody knows who you are and where you come from. The minute you pick up the phone and somebody says, what’s your postcode, they can tell you where you live and who you are. So, what’s the difference?

To justify their concerns about surveillance, interviewees also drew on information they had about various media scandals. For example, one interviewee drew on the Snowden revelations as an example of why not to trust governments;

I have heard about these ideas [about apps]. I find it deeply uncomfortable. Not in principle, but just because our governments have a terrible track record of using that data responsibly. Just thinking back to the Snowden revelations, what came out about GCHQ [government communications headquarters], which is our UK intelligence-gathering institution. And I just have no trust in them to use it responsibly (interviewee 21).

In contrast, interviewee 11 spoke about the growing controversy of social media conglomerate Facebook in public life, and in doing so, subsumed their concerns about contact tracing apps with other concerns surrounding private corporations’ tracking more generally. They used this to draw conclusions about the surveillance capacity of these apps: *‘you go into downloading apps, hoping for the best intentions, but Facebook has taught us otherwise. These concerns linger at the back of my mind’.*

Other interviewees took a different perspective on the use of contact tracing apps in terms of being ‘tracked’. For them, there was a resignation that despite their hard work to protect liberties, individuals were still being tracked, or could still bme a message everye tracked, by both the private sector and the UK government *(‘I’d prefer if they didn’t keep track of me. But … they can probably already do it’* (interviewee 20). There was such a growing perception of being tracked, particularly by private companies, that there were hints in interviewees’ discussions that this resignation at times, perhaps shifted to an almost comfortableness; *‘Google maps track my phone … .it sends me a message every month … about restaurants and Christ knows what… .It’s the world we live in’* (interviewee 10). In fact, interviewee 14, who worked in information technology, was not just resigned to being tracked, but seemingly unconcerned because of the lack of direct impact on their life that they were aware of:

we could talk forever and a day about … the big corporate IT world and how it’s used by Government and uses our data. It doesn’t really make any odds to me … in the long run I get on with my life.

This tracking was viewed as part of changing times, increased technology use and big data, and the political neoliberal climate in which we now live (‘we *live in a world where you either get with it or you get left behind. You can’t do much’* (interviewee 11)), and for these interviewees, the use of contact tracing apps should be seen in this context.

Finally, those supportive of using an app explained away concerns about surveillance by noting that the apps were a short-term measure, that it was for the ‘common good’ and that, perhaps, it was their ‘naivety’, which led them to not worry about Big Brother (‘I *can imagine that there would be a backlash against that, because it’s kind of Big Brother’* (interviewee 3); *‘I’m a bit naïve in the fact that I’m quite happy for Big Brother to do all these things …. for the common good’* (interviewee 27)).

### Balancing risk and benefits: the role of (dis)trust in the government

The surveillance, privacy and data protection concerns mapped above by those interviewees who were less supportive of digital contact tracing apps often correlated with their broader distrust in the UK government to store and analyse the data collected by an app responsibly: *‘I’d be very nervous about the government having a central database with all this data in it’* (interviewee 6). For instance, interviewee 17 emphasised the UK government’s poor track record with responsible data management procedures, and used this to highlight their lack of faith in the government to develop, implement and use an app appropriately:

you’re talking about undoing years of deceit and mismanagement. I don’t think you can just make a policy announcement and say, look, guys, we’re going to do it properly this time, trust us. I’m not going to buy it.

In fact, interviewees drew on a range of specific instances of poor data governance that they had come across in the media – both in the UK and internationally – and used these examples to justify their views about the use of digital contact tracing apps. Interviewee 21, for example, drew on the Snowden revelations, in which classified information from the United States (US) national security agency was leaked with wide-ranging international consequences, to emphasise their concerns about responsible governance of data in general. From this they questioned their trust in the UK government, and by extrapolation, the use of digital tracing apps:

I find it deeply uncomfortable. Not in principle, but just because our governments have a terrible track record of using that data responsibly. Just thinking back to the Snowden revelations … I just have no trust in them to use it responsibly.

In another interview, interviewee 31 drew on the political context of Brexit to emphasise their lack of trust in the government, and by association their concern about the development of digital contact tracing apps. This interviewee was anxious that post-Brexit, the UK would move towards a ‘less robust’ data security infrastructure – one that is much more aligned with that of the US:

I’m very sceptical at the moment of anything that comes out of the NHS and the government, as to what it will be used for …. We’re leaving the EU (European Union), so our data sharing and data security standards will completely bomb because … we won’t still continue to co-opt the European system, we will use the American system where security and sharing of data is far less robust.

It was perhaps because of this lack of trust in the government, as well as interviewees’ perceptions of the risks associated with using an app, that some interviewees’ spoke about needing *‘more reassurance’* (interviewee 17) about how the data would be stored and processed before they would contemplate using any such app. Interviewees wanted more clarity and information about any potential UK app, and the data protection mechanisms that would be in place. Without this, it made it difficult for interviewees, who felt ‘conflicted’ (interviewee 5), to decide if they should support the use of any potential UK contract tracing app or not: ‘I *think that if we could be given assurances that say the data would be destroyed once the pandemic was well and truly over, I don’t really know …* (interviewee 5).

## Discussion

Corroborating other research (AdaLovelace Institute, 2020a; Williams et al., 2020), interviewees had a range of views about the use of digital contact tracing apps as part of the UK’s response to the COVID-19 pandemic. This ranged from those who were very supportive of using apps for contact tracing and spoke in terms of solidarity about using the data for research purposes for the benefit of all, to those who were extremely cautious about digital contact tracing apps. Here, interviewees framed their concerns around their distrust in the UK government, in terms of privacy and surveillance issues, and in terms of issues relating to the growing and expanding ‘Big Data’ landscape to which the interviewees felt increasingly resigned. These were operationalised by identifying with recycled British national narratives associated with individual liberty, and expressions and language borrowed from author George Orwell, which have often featured prominently in British culture as a warning against the erosion of enshrined civil and individual liberties.^4^

While not explicitly mentioned by our interviewees, some of our interviewees’ growing and pronounced concerns for privacy, anonymity, and data protection might reflect a series of recent controversies and ‘Big Data disasters’ (McDonald, 2016) involving partnerships with the UK government and Big Data corporations, which have been said to have depleted trust in the UK government to handle NHS data responsibly and according to the social licence that has been created between the government and society (Lucassen et al., 2017). These have included the Google DeepMind, London Royal Free scandal, which involved the transfer of identifiable patient records across the entire Trust (Powles & Hodson, 2017), and the 2014 public relations failure of care.data – an initiative that aimed to improve the use of GP data for research but received harsh public criticism (Public Health Research Data Forum, 2015). They might also have reflected recent shock events including the 2013 Snowden revelations, the 2016 Brexit Referendum,^5^ and the 2018 Cambridge Analytica scandal, which have brought to the forefront new concerns over the opacity of many digital surveillance practices and technologies (Roberts, 2020b), and have highlighted further the growing authority of Big Tech corporations and ‘Big Data’ processing firms to assist governments in the collection, production, and presentation of publicly produced data for security purposes (Roberts, 2019). In fact, some of our interviewees specifically drew on some of these shock events in their discussions about apps.

Despite the aforementioned issues, we cannot know whether those interviewees’ who expressed concerns about digital contact tracing apps would then choose not to download an app once it had been developed by NHSX (or by any of the devolved nations (Scotland, Wales, Northern Ireland) that have chosen not to use the NHSX app). This is especially true if we locate our findings in the broader context of general UK support for using digital contact tracing apps (Abeler et al., 2020; Duffy, 2020). In fact, to date, following the re-development of the NHSX app and its launch in September 2020, it has been downloaded 20 million times.

Nevertheless, and while the UK contact tracing app is already implemented, we can draw from our findings as they relate to responsible innovation for emerging public health intervention technologies in two ways. First, the way in which our interviewees drew on a range of individual experiences and social factors to construct ideas about the app is a reminder of the importance of considering the heterogeneity of factors that affect public views associated with emerging public health technologies. Here we showed how the wider political climate at the time of the first lockdown, including a number of recent big data scandals, was relevant to public views. In future, this needs to be considered, and specifically the socio-political climate within which the public receive information about public health interventions. As our findings have shown, interviewees associated their beliefs about the app much more broadly than just a public health intervention, or indeed health more generally, and by understanding what matters to individuals, we can better integrate these views into an anticipatory governance process that considers and responds to them.

Second, our findings point to the type of concerns an anticipatory governance might have (and in the future could) consider. Our findings identified interviewees’ worries of using public health-purposed contact tracing apps on both governmentality and civil liberties. Such concerns may be grounded (Kitchin, 2020; Wienroth et al., 2020), for example, recently we have seen how Singaporean officials have not ruled out that data collected through the Singapore contact tracing app may be used for criminal investigations (Taylor, 2021)– and this is an aspect that would need to be considered during anticipatory governance. However, the way at least some of the interviewees constructed these concerns appeared to reflect their misunderstandings about the capabilities and functionality of digital contact tracing generally, and a potential UK app, specifically (Williams et al., 2020). For instance, many interviewees’ narratives resonated with the geo-location style mandatory tracking that has been used in other countries such as China, Taiwan and South Korea, which was ruled out early on in the development of the NHSX app because of privacy concerns. Interviewees’ responses perhaps reflected different snippets of information they had heard about COVID-19 apps in the media more generally. Interviewees’ narratives also suggested that it was Big Tech corporations (including Apple and Google) that were directly tracing individuals via their smartphones and GPS locations, rather than, in actuality, providing the underlying infrastructure for digital contact tracing for apps approved by national states to launch these digital programmes. These misunderstandings raise questions about the public health implications of such misunderstandings, particularly around the need for clearer public information on the design and parameters of contact tracing apps (Williams et al., 2020). For some, time and resource constraints caused by the COVID-19 pandemic, along with the public health emergency, justified the prioritisation of the tracing app development over public communication and engagement, despite the importance of effective communication and engagement in maintaining public support and trust. However, failure to engage with, and take seriously public concerns with the expansion and intensification of surveillance practices during a period of a national public health emergency represents a missed opportunity to foster a pluralistic democratic deliberation on these issues through anticipatory governance.

As a final point, interviewees reflected on the need to balance invasions of privacy/personal autonomy through new digital surveillance measures on one hand, and the detection and containment benefits for public health on the other. Interviewees’ balancing of invasions of privacy versus benefits is interesting because of its polarisation of issues, and in terms of the way moral decisions are constructed. Viewing moral decisions in this way, that is, as polarised trade-offs between two competing values, omits a range of other factors that need to be taken into account (Goldenfein et al., 2020; Pavone et al., 2016). For example, even when digital technologies are used, there are many technological and ethical solutions to data collection, access and use that could ensure the responsible development of digital contact tracing. For instance, evidence from Taiwan’s responses to COVID-19 demonstrates how new digitised health surveillance practices, including contact-tracing apps, can be operationalised as part of larger health security responses in a method which is also compatible with respect for personal privacy and autonomy, and within contexts where public trust in government and institutions are established and robust (Wang et al., 2020). Samuel and Prainsack (2019) suggest a better way to consider moral issues is on a balance scale, where various weights need to be placed on either side of the scale, representing the diversity of ethical and social issues that are associated with the use of a technology. Without considering all of these balancers in the scale, there is a possibility that the debate will be narrowed, and this was indeed present in our findings.

In conclusion, our interviews with members of the public showed how the current socio-political climate, along with other factors, played a role in influencing interviewees’ views about contact tracing apps, particularly around issues of privacy and surveillance. We have shown that situating these findings in a broader framework of public engagement for responsible innovation requires us to confront and respond to these findings through an anticipatory governance process. More broadly, an anticipatory responsible innovation framework might be a useful approach to adopt for other emerging public health technological innovations.

### Limitations

There are a number of methodological limitations to this study. First, while some interviewees were from Scotland, Wales, and North England, the majority of participants were from South England. Second, selfreported ethnicity of interviewee participants was not collected as part of the SolPan project so we cannot determine the demographic spread of our participants. Third, our findings represent a snapshot of views about contact tracing apps very early in NHSX’s development of the technology, and so misunderstandings are likely common, and views reflect hypothetical decisions about app use. Lastly, while this article has worked to present and account for the diversity of perceptions and understandings of digital contact tracing practices during a particular phase of the ongoing COVID-19 global pandemic in the UK, it additionally acknowledges the highly dynamic and rapidly shifting developments in contact tracing initiatives. While research, findings and attitudes to contact tracing technologies continue to transform and evolve, the concerns articulated by respondents within this UK study, particularly centered around concepts of transparency, privacy and cultures of trust in public authorities during public emergencies remain foundational to ongoing global analyses of contact tracing practices both during and after the cessation of public health emergencies.

## Figures and Tables

**Table 1:** Self-reported demographic characteristics of interviewees.

*Characteristic*	Number of participants (%)
*Gender*	
Male	20 (57)
Female	14 (40)
*Age*	
18-30	6 (17)
31-45	11 (31)
46-60	11 (31)
61-70	5 (14)
70 plus	2 (6)
*Employment status*	
Employed (long-term contract)	17 (49)
Employed (short-term contract)	2 (16)
Self-employed	5 (14)
Unemployed	5 (14)
Retired	5 (14)
Other	2 (16)
*Highest level of education*	
Less than 10 years	2 (6)
10-14 years (e.g. High school Diploma)	10 (29)
Higher Education	23 (66)

## Notes

1 Reports suggest at least 60% of the population need to be using the app to ensure it works efficiently.

2 API stands for Application Programming Interface and constitutes the underlying architecture that allows smartphones to exchange data with a certain app and with each other.

3
https://digigov.univie.ac.at/solidarity-in-times-of-a-pandemic-solpan/

4 For example, see https://bigbrotherwatch.org.uk/

5
https://www.kcl.ac.uk/policy-institute/research-analysis/the-publics-brexit-misperceptions
